# Supplementary material for: Sphingomonas sediminicola Dae20 Is a Highly Promising Beneficial Bacteria for Crop Biostimulation Due to Its Positive Effects on Plant Growth and Development
Source: Microorganisms. 2023 Aug 11;11(8):2061. doi: 10.3390/microorganisms11082061 (PMC10459697; doi:10.3390/microorganisms11082061)
Supplement: Supplementary file 1 [file microorganisms-11-02061-s001.zip › microorganisms-2540518-supplementary.pdf]

Supplementary Materials:

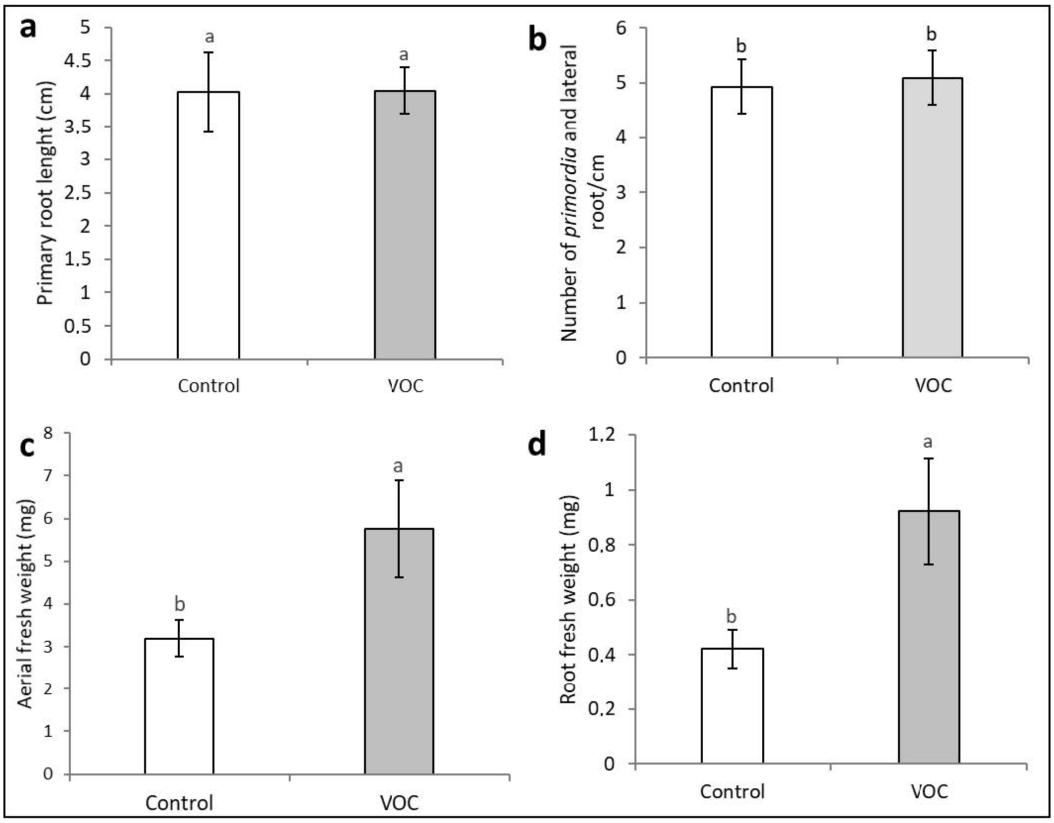

Figure S1. Primary root (a), primordia number and lateral root density (LR) (b), shoot fresh weight (c) and root fresh weight (d) in 10-day-old *Arabidopsis thaliana* in the presence or not (control) of volatile organic compounds (VOC) released by *S. sediminicola*. Error bars represent SD. Letters represents a significant difference from the control. Statistical analysis is Anova test.

Table S1. Primers used to target specific gene sequences in *Arabidopsis thaliana* inoculated and not-inoculated with *S. sediminicola*.

	Target	Left primer	Right primer
Auxin	*GH3.5*	GTTGTCACAAATTTCGCTGGCTTG	GCGCGTTGTTGTAGAAACCAGTC
	ARF7	TCAAGGTCACAGTGAGCAAGTCG	TGTGGAGCATGCATATGAGCTTGG
	ARF19	ACAGCTCGAAGATCCGCTAACC	TGCACGCAGTTCACAAACTCTTC
Abscisic acid	*ABI1*	CATGTCGAGATCCATTGGCGATAG	ACTCTCTTCACAGCCGTCACTTC
Jasmonic acid	*LOX2*	TGGAGGGCATAACTTGGTCGAG	TGCGTAGTCTTCTACCGTAATCCG
	JAZ1	TGTTCTGAGTTCGTCGGTAGCC	AGTTCCATTGACATCAGGCTTGC
	PDF1.2	CTTGTTCTCTTTGCTGCTTTCGAC	TTGGCTCCTTCAAGGTTAATGCAC
Salicylic acid	*MYC2*	AACCACGTCGAAGCAGAGAGA	TTGGTACAACCGCTCGTAACGC
	PR1	ACACGTGCAATGGAGTTTGTGG	TTGGCACATCCGAGTCTCACTG
	EDS1	GCTCAATGACCTTGGAGTGAGC	TCTTCCTCTAATGCAGCTTGAACG
	ACTIN2	TCTTCCGCTCTTTCTTTCCAAGC	ACCATTGTCACACACGATTGGTTG
